# Perioperative Management of Lewis-Sumner Syndrome

**DOI:** 10.7759/cureus.36297

**Published:** 2023-03-17

**Authors:** Filipa Sales, Ana Raquel S Cruz, Filipa Maldonado, Mariana Cunha

**Affiliations:** 1 Anesthesiology, Hospital Pedro Hispano, Matosinhos, PRT

**Keywords:** anesthetic management, neuromuscular blockers, demyelinating neurological disorder, chronic inflammatory demyelinating polyneuroradiculopathy (cidp), lewis sumner syndrome

## Abstract

Lewis-Sumner syndrome (LSS) is a rare immune-mediated neuromuscular disorder. It shares some clinical and pathological features with chronic inflammatory demyelinating polyneuropathy (CIDP). We report on the anaesthetic management of a patient with LSS. There are several concerns when anaesthetizing patients with demyelinating neuropathies, the main ones being the post-operative worsening of symptoms and respiratory depression related to muscle relaxants. In our experience, the rocuronium effect was prolonged and an even lower dosage (0.4 mg/kg) was sufficient for intubation and maintenance. Sugammadex allowed total reversion of neuromuscular block and no respiratory complications occurred. In conclusion, the combination of lower dose rocuronium and sugammadex was safely used in a patient with LSS.

## Introduction

Lewis-Sumner syndrome (LSS) is a rare immune-mediated neuromuscular disorder, specifically an acquired demyelinating polyneuropathy, with a prevalence between one and nine out of 1,000,000 [1]. It is characterized by an adult-onset asymmetrical distal weakness of the upper or lower extremities and motor dysfunction. This syndrome is also called multifocal acquired demyelinating sensory and motor (MADSAM) neuropathy and it is a variant of chronic inflammatory demyelinating polyneuropathy (CIDP) [2].

There is very limited information available in the medical literature on the perioperative management of patients suffering from CIDP. To our knowledge, there are no published reports on the anesthetic management of patients with this variant. We report on the anesthetic management of a patient with LSS, who presented for lumbar discectomy in October 2019.

A poster about this case was presented online at the Euroanaesthesia Congress in 2020. This report contains further information, discussion, and follow-up findings.

## Case presentation

A sexagenarian, American Society of Anesthesiologists (ASA) III, female with diabetes presented for L4-L5 laminectomy, foraminotomy, and discectomy due to a herniated disk with significant lumbar stenosis. She had a previous diagnosis of LSS with severe degenerative lumbosacral polyradiculopathy and sensory-motor polyneuropathy.

A pre-anesthetic evaluation with an emphasis on neurological deficits was performed. The patient was tetraparetic, with a limb muscle strength of 4/5 (Medical Research Council (MRC) scale). She was able to walk very short distances without support but used two crutches for most of her daily activities. Another important aspect was the need for chronic urinary catheterization due to neurogenic bladder. Chronic medication was azathioprine 150 mg once daily, pregabalin 150 mg twice daily, and oral antidiabetics.

Surgery was performed under general anesthesia with standard monitoring, including invasive blood pressure, bispectral index, and neuromuscular monitoring with train-of-four (TOF) count. General anesthesia was induced with fentanyl, propofol, and rocuronium (0.4 mg/kg) and maintenance was assured with total intravenous anesthesia (TIVA). Arterial blood gas analysis was performed 30 minutes after general anesthesia induction (Table 1).

**Table 1:** Intraoperative arterial blood gas analysis FiO_2_: fraction of inspired oxygen; pCO_2_: partial pressure of carbon dioxide; pO_2_: partial pressure of oxygen; Na+: sodium ion; HCO_3_: bicarbonate; SO_2_: oxygen saturation; Hb: hemoglobin

Volume Controlled Invasive Mechanical Ventilation - FiO_2_ 35%		
Parameter	Result	Reference range
pH	7.49	7.35-7.45
pCO_2_	38 mmHg	35-45 mmHg
pO_2_	208 mmHg	80-100 mmHg
Na+	134 mmol/L	135-145 mmol/L
K+	3.2 mmol/L	3.5-5.5 mmol/L
HCO_3_-	29 mmol/L	22-26 mmol/L
SO_2_	100%	90-100%
Hb	9 g/dL	12-14 g/dL

The initial rocuronium bolus was sufficient to provide intubation conditions (TOF count zero) in under two minutes and no further administrations were necessary throughout the surgery, which lasted 110 minutes. Before emergence, the TOF ratio was 49, so the neuromuscular block was reversed with 2 mg/kg of sugammadex. Complete reversal was rapidly achieved after the administration with a TOF ratio of 90% and the patient was extubated.

Blood gas analysis performed in the recovery room with fraction of inspired oxygen (FiO_2_) of 28% showed no respiratory insufficiency (Table 2).

**Table 2:** Postoperative arterial blood gas analysis results FiO_2_: fraction of inspired oxygen; pCO_2_: partial pressure of carbon dioxide; pO_2_: partial pressure of oxygen; Na+: sodium ion; HCO_3_: bicarbonate; SO_2_: oxygen saturation; Hb: hemoglobin

Spontaneous Ventilation - FiO_2_ 28%		
Parameter	Result	Reference range
pH	7.48	7.35-7.45
pCO_2_	39 mmHg	35-45 mmHg
pO_2_	153 mmHg	80-100 mmHg
Na+	130 mmol/L	135-145 mmol/L
K+	3.2 mmol/L	3.5-5.5 mmol/L
HCO_3_-	29 mmol/L	22-26 mmol/L
SO_2_	99%	90-100%
Hb	8.7 g/dL	12-14 g/dL

The patient remained stable throughout the postoperative period without worsening of sensory-motor deficits. She started ambulation the day after surgery and was discharged home three days later. On her neurological follow-up, two weeks after surgery, she reported pain relief and better mobility of the lower limbs, needing only one crutch.

Two years later, the patient had no additional deficits but maintained a muscle strength of 4/5 on hip flexion and preferred to walk with two crutches, for balance.

## Discussion

Given that there is no available information on the anesthetic management of LSS, most of our research was focused on CIDP. We extrapolated our findings to LSS, since it is a variant and shares some pathophysiologic features.

There are several concerns when anesthetizing patients with demyelinating neuropathies, the main ones being the post-operative worsening of symptoms and respiratory depression related to muscle relaxants. In order to establish a reliable baseline, it is of the utmost importance to collect a detailed patient history, conduct a thorough neurological exam, and register the findings.

Although there is a case report by Gupta et al. describing the safe use of regional anesthesia in a patient with CIDP [3], we were concerned with neurologic monitoring in the immediate postoperative period, seeing as the patient was being submitted to a lumbar discectomy and could be at increased risk for complications due to LSS.

There has also been some apprehension regarding the use of muscle relaxants in patients with neuromuscular disorders due to their increased sensitivity to non-depolarizing neuromuscular blockers and the risk of hyperkalemia with succinylcholine. In fact, Takekawa et al. opted to avoid these drugs altogether and resort to a combination of general and regional anesthesia for a CIDP patient undergoing a Hartmann procedure [4].

The use of succinylcholine is particularly concerning due to the possibility of life-threatening hyperkalemic responses in patients with demyelinating neuropathies, as described by Levine and Brown in a patient with multiple sclerosis [5]. In 2010, Hor reported a case of cardiac arrhythmia following succinylcholine administration in a patient with Guillain-Barré syndrome (GBS) [6], and four years later, Raja and Waheed reported a hyperkalemic cardiac arrest following its administration even after resolution of clinical symptoms of GBS. [7] These exacerbated hyperkalemic responses are thought to be caused by the upregulation of the nicotinic-acetylcholine receptor on skeletal muscle with an increase in extra junctional acetylcholine receptors [8,9]. Surprisingly, a case series by Mortenson et al. in CIDP patients found no obvious cases of hyperkalemia in five patients who received succinylcholine, although they did not check serum potassium levels or electrocardiographic signs of hyperkalemia [10].

Regarding non-depolarizing agents, Pogson et al. described a case in which vecuronium 0.11 mg/kg produced prolonged neuromuscular blockade in a patient with Charcot-Marie-Tooth neuropathy, although there are conflicting reports concerning the response to neuromuscular blocking drugs in these patients [11]. In GBS, the response to nondepolarizing muscle relaxants may vary depending on the phase of the disease with resistance in the denervated phase, followed by increased sensitivity during the reinnervation phase. [8] As for CIDP, Hara et al. reported the prolonged effect of vecuronium in a patient suffering from the disease and suggested that muscle relaxants should either not be used or be used with smaller doses [12].

In the current case, neuromuscular blockade was preferred by the surgical team to facilitate surgical technique and, given the information gathered from case reports on CIDP, we considered it to be a reasonable course of action in LSS.

We opted for rocuronium due to its fast onset and absence of active metabolites and, most importantly, the existence of a specific reversal agent. The combination of rocuronium (0.6-0.7 mg x kg(-1)) and sugammadex has been successfully used by Maruyama and colleagues in 2015 in two patients with CIDP, but not in LSS [13]. The choice of TIVA instead of volatile anesthetics for maintenance was due to the fact that the latter enhances the neuromuscular blockade produced by nondepolarizing muscle relaxants [14].

In our experience, the rocuronium effect was prolonged and an even lower dosage (0.4mg/kg) was sufficient for intubation and maintenance. Sugammadex allowed total reversion of neuromuscular block and no respiratory complications occurred.

## Conclusions

The combination of lower dose rocuronium and sugammadex was safely used in a patient with LSS. Although it would be ideal to have further studies regarding the choice of neuromuscular blocking agent in such patients, it is unrealistic to expect high-quality evidence on such a rare disease. This case may serve as an example of a safe approach and guide fellow anesthesiologists on managing patients suffering from LSS.

## References

[1] (2023). Orphanet: Lewis-Sumner syndrome. https://www.orpha.net/consor/cgi-bin/OC_Exp.php?lng=EN&Expert=48162.

[2] Lehmann HC, Burke D, Kuwabara S (2019). Chronic inflammatory demyelinating polyneuropathy: update on diagnosis, immunopathogenesis and treatment. J Neurol Neurosurg Psychiatry.

[3] Gupta B, Agrawal P, D'souza N, Sawhney C (2011). Anaesthetic management and implications of a case of chronic inflammatory demyelinating polyneuropathy. Indian J Anaesth.

[4] Takekawa D, Nakai K, Kinoshita H, Saito J, Kitayama M, Kushikata T, Hirota K (2019). Anesthetic management of a patient with chronic inflammatory demyelinating polyneuropathy by combination of total intravenous and regional anesthesia. JA Clin Rep.

[5] Levine M, Brown DF (2012). Succinylcholine-induced hyperkalemia in a patient with multiple sclerosis. J Emerg Med.

[6] Hor JY (2010). Cardiac arrhythmia after succinylcholine administration in a patient with Guillain-Barré syndrome--a case report. Middle East J Anaesthesiol.

[7] Raja W, Waheed S (2014). Cardiac arrest after succinylcholine administration in a patient recovering from Guillain-Barre syndrome. J Coll Physicians Surg Pak.

[8] Romero A, Joshi GP (2013). Neuromuscular disease and anesthesia. Muscle Nerve.

[9] Martyn JA, White DA, Gronert GA, Jaffe RS, Ward JM (1992). Up-and-down regulation of skeletal muscle acetylcholine receptors. Effects on neuromuscular blockers. Anesthesiology.

[10] Mortenson AR, Sprung J, Watson JC, Dyck PJ, Weingarten TN (2017). Chronic inflammatory demyelinating polyradiculoneuropathy and anesthesia: a case series. Acta Neurol Belg.

[11] Pogson D, Telfer J, Wimbush S (2000). Prolonged vecuronium neuromuscular blockade associated with Charcot Marie Tooth neuropathy. Br J Anaesth.

[12] Hara K, Minami K, Takamoto K, Shiraishi M, Sata T (2000). The prolonged effect of a muscle relaxant in a patient with chronic inflammatory demyelinating polyradiculoneuropathy. Anesth Analg.

[13] Maruyama N, Wakimoto M, Inamori N, Nishimura S, Mori T (2015). Anesthetic management of three patients with chronic inflammatory demyelinating polyradiculoneuropathy (Article in Japanese). Masui.

[14] Paul M, Fokt RM, Kindler CH, Dipp NC, Yost CS (2002). Characterization of the interactions between volatile anesthetics and neuromuscular blockers at the muscle nicotinic acetylcholine receptor. Anesth Analg.

